# Degradation enhancement of rice straw by co-culture of *Phanerochaete chrysosporium* and *Trichoderma viride*

**DOI:** 10.1038/s41598-019-56123-5

**Published:** 2019-12-23

**Authors:** Kai-Jian Chen, Ji-Chao Tang, Bao-Hong Xu, Shi-Le Lan, Yankun Cao

**Affiliations:** 1 College of Animal Science and Technology, Hunan Agricultural University, Changsha, 410128 China; 2 College of Bioscience and Biotechnology, Hunan Agricultural University, Changsha, 410128 China; 3 R&D Center, Guangdong Meilikang Bio-Science Ltd., Dongguan, 523808 China

**Keywords:** Environmental biotechnology, Applied microbiology, Fungi

## Abstract

Straw is one of the most abundant stock of renewable biomass from crop production. However, its utilization efficiency is still very low. Although co-cultivation of fungi increases the degrading rate, the co-cultivation condition needs to be optimized. To optimize the co-culture condition of *Phanerochaete chrysosporium* and *Trichoderma viride* degrading rice straw, we first tested the antagonistic characteristic between the fungi. The results showed that the best co-culture pattern was to first inoculate *P. chrysosporium* and culture for 4 days, then inoculate *T. viride*, and co-culture the two fungi for 4 days. The optimum fermentation condition was 14% (w/v) of inoculum concentration, the equivalent inoculation of the fungi, culture temperature at 30 °C, and 1:1.4 for solid-liquid ratio. Under the optimum condition, the degradation ratios of lignin and cellulose were 26.38% and 33.29%, respectively; the soluble carbon content in the culture product was 23.07% (w/v). The results would provide important reference information for the efficient utilization of rice straw to produce more accessible energy resources, such as ethanol and glucose.

## Introduction

Straw is one of the most abundant stock of renewable biomass by-products in the crop production such as wheat, rice, and corn, and exhibits huge potentiality to be converted to “green energy”^1,2^. The main components of the straw of most crops are cellulose, hemicellulose, and lignin. Cellulose is the most abundant component in the straw and a renewable resource, as a raw material for fuels, chemicals, and food industries^1^. Therefore, most studies of biodegradation and reutilization of straws focus on cellulose degradation, and a lot of microbiomes have been identified as cellulose degrading agency, such as *Neurospora crassa*, *Trichoderma viride*, and brown-rot fungi^3,4^. However, the degradation ratio and efficiency of straw cellulose are still very low^1^.

Chemical constitution analysis shows that lignin forms a matrix surrounding the cellulose in woody cell walls, which protects the hemicellulose and cellulose from microbial depolymerization^1,5^. Decomposition of the lignin changes the complex structure of lignin and cellulose with hemicellulose, and exposes cellulose and hemicellulose to cellulase. Therefore, in order to efficiently utilize the energy of the straw, a process called delignification is required to break down the lignin structure and provide an access for enzymatic saccharification of the cellulosic component^2^.

There are various methods to break down the lignin structure in the straw, such as the thermal treatment^1^, chemical degradation^6^, and biological degradation^7,8^. Biological degradation has received extensive attention as it is easily integrated with subsequent processes of cellulose and hemicellulose degradation. Rees *et al*.^9^ reported that co-culture with the hydrogen-utilizing acetogenic bacterium *Acetitomaculum ruminis* increased acetate production of *Neocallimastix patriciarum*, *Neocallimastix* sp. L2, or *Methanobrevibacter smithii*. However, co-culture does not just mechanically integrate two metabolic reactions in the same reactor, but needs to regulate and advance to each other of the co-cultivated microorganisms. Their growth requirement also needs to balance.

White-rot fungi are among the best degrading agents for lignin^5^. White-rot fungi can produce lignin peroxidase, lemanganese peroxidase, and laccase at low-glucose medium, which are the major oxidoreductases of lignin^10,11^. In many studies, rice straws were degraded by white-rot fungi and *T. viride*. Currently, co-cultivation of white-rot fungi and *T. viride* to degrade rice straw has also been reported^12,13^. However, co-cultivation patterns and conditions still need to be optimized as the degradation ratios of lignin and cellulose are still very low.

In the present study, we utilized *T. viride* ACCC30169 and white-rot fungus *Phanerochaete chrysosporium* ACCC30942 to screen the optimum co-culture condition of rice straw through antagonistic experiment and co-culture experiments. The results would provide important reference information to utilize rice straw to efficiently produce available resources, such as easily available forage and energy.

## Results and Discussion

### *T. viride* overgrown than *P. chrysosporium* in plates

**Figure 1:**
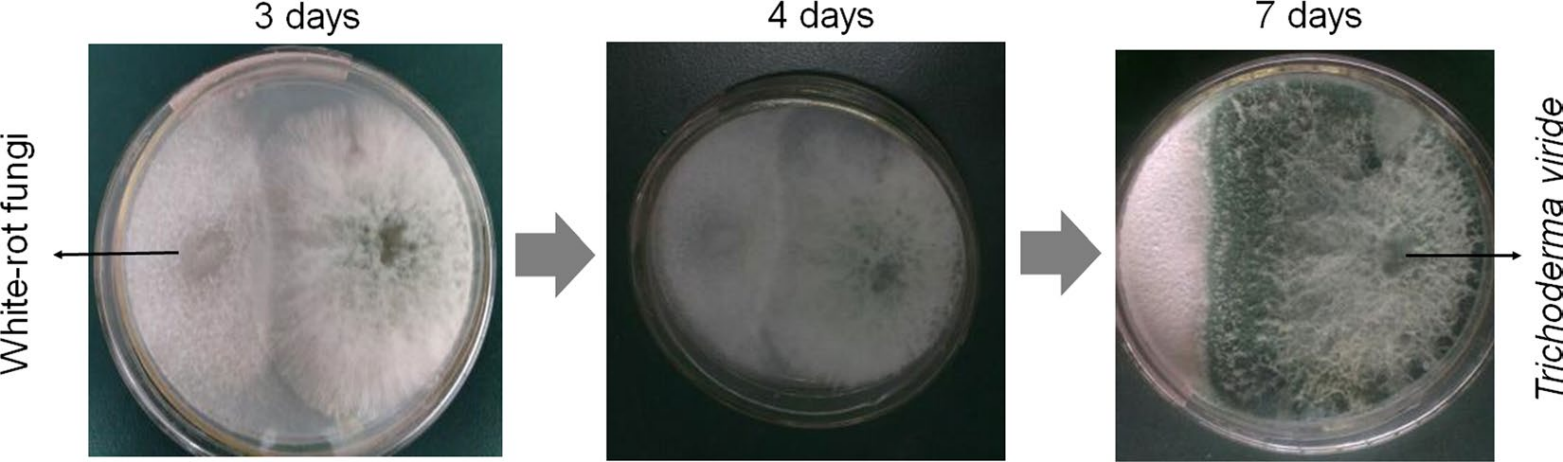
Antagonistic tests between *P. chrysosporium* and *T. viride* in PDA solid plate medium.

To analyze whether the fungi suit to simultaneous culture, we firstly tested the antagonistic characteristic between the fungi. *T. viride* overgrown than *P. chrysosporium* in plates on the fourth day. *T. viride* overwhelmingly overgrown than *P. chrysosporium* on the seventh day (Fig. 1). The results implied the two fungi did not suit to co-culture synchronously. As *T. viride* overgrown than *P. chrysosporium*, we predicted that successive inoculations of *P. chrysosporium* and *T. viride* probably raised the degradation efficiency to rice straw. In addition, considering white-rot fungi, such as *P. chrysosporium*, are among the best degrading agents for lignin^5^, we predicted that *P. chrysosporium* should be firstly inoculated to the culture medium.

### First inoculation of *P. chrysosporium* and culture for 4 days, followed by inoculation of *T. viride* and then co-culture (B4-L) was the optimum co-culture pattern

**Figure 2:**
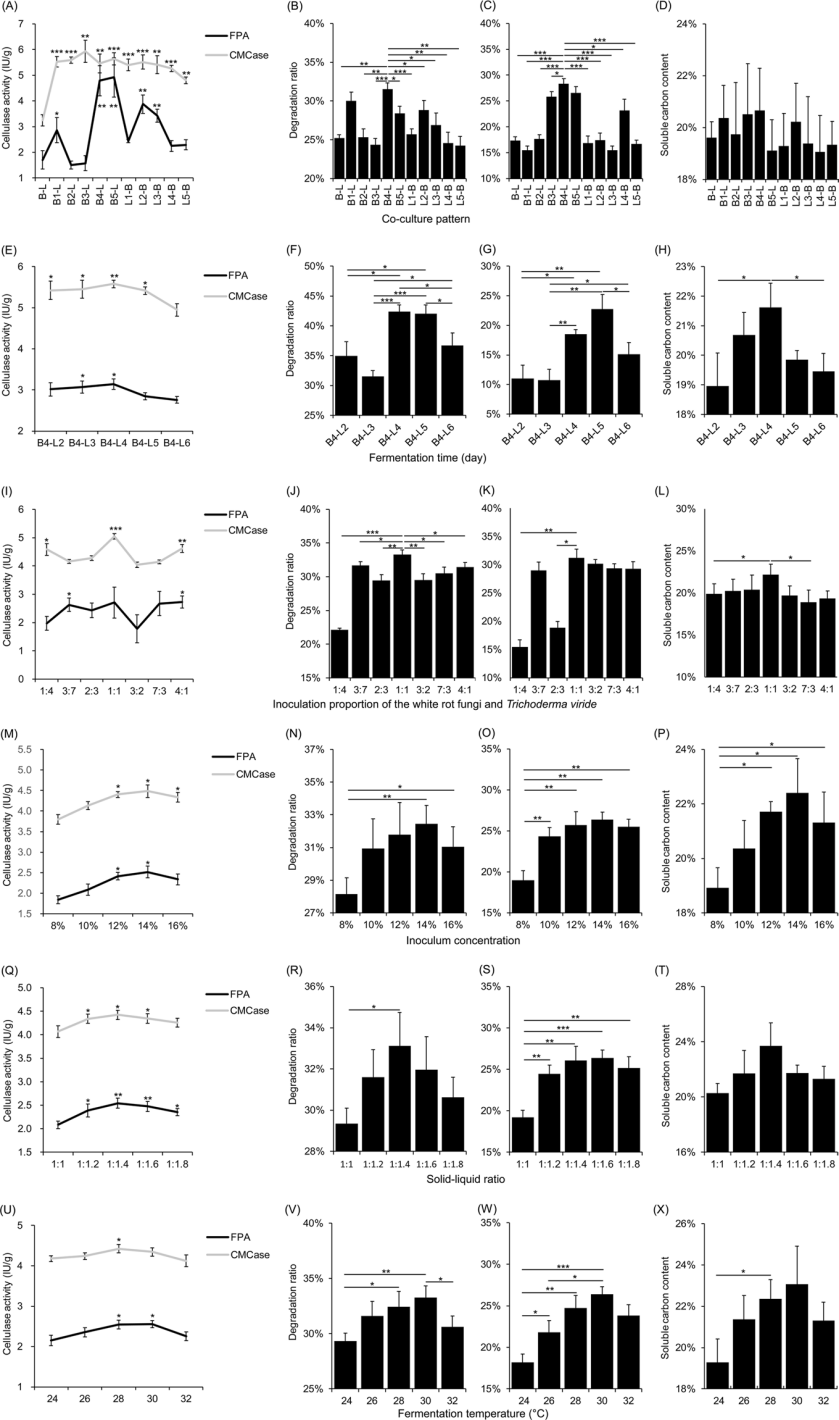
Fermentation effects of *P. chrysosporium* and *T. viride* co-culture under different conditions. (**A,E,I,M,Q,U**) showed the changes of cellulase activities of different treatment groups. (**B,F,J,N,R,V**) showed the degradation ratios of cellulose; (**C,G,K,O,S,W**) showed the degradation ratios of lignin; and (**D,H,L,P,T,X**) showed the soluble carbon contents in the culture product. For panels (**A–D**), the total culture time was 7 days. The different co-culture patterns were named by the firstly inoculated fungus (B represented *P. chrysosporium* and L represented *T. viride*) and cultured days (from 1 to 5), and the secondly inoculated fungus. For instance, B2-L represented that firstly inoculated *P. chrysosporium* and cultured 2 days, and then inoculated *T. viride* and co-cultured 5 days. B-L represented that inoculated *T. viride* and *P. chrysosporium* synchronously and co-cultured 7 days. For panels (**E–H**), the optimum co-culture time patterns were named by the firstly cultured 4 days after inoculating *P. chrysosporium* (B4), and then co-cultured days (from 2 to 6) after inoculating *T. viride* (L2 to L6). For instance, B4-L3 represented the firstly inoculated 4 days of *P. chrysosporium*, and then inoculated *T. viride* and co-cultured 3 days. (A), the statistically significant marker “*” shows the different significances between (**B–L**) and other co-culture patterns. (**B,C**), just show the significant differences between B4-L and other co-culture patterns. (**E**), the statistically significant marker “*” shows the different significances between B4-L6 and other co-culture time patterns. (**I**), the statistically significant marker “*” shows the different significances between group 3:2 and other groups. (**M,Q**,**U**), the statistically significant marker “*” shows the different significances between the first group and other groups. (**J**) just show the significant differences between group 1:1 and other co-culture groups. *p < 0.05; **P < 0.01; ***p < 0.001.

The cellulase activity in co-culture pattern B-L was significantly lower than other treatment groups (Fig. 2A). This result implied that growth of *P. chrysosporium* was probably competitively depressed by *T. viride*. However, the cellulase activity of the co-culture patterns inoculated *T. viride*and first and cultured for 1 to 5 days, and then inoculated *P. chrysosporium* were also higher than that of the simultaneously inoculated *P. chrysosporium* and *T. viride* (Fig. 2A). The cause of this phenomenon is unknown yet. Degradation ratios of lignin (DRL), degradation ratios of cellulose (DRC), and soluble carbon content (SCC) in the culture product of the co-culture pattern B4-L, i.e. first inoculation of *P. chrysosporium* and culture for 4 days, followed by inoculation of *T. viride* and then co-culture for 3 days, were the highest (Fig. 2B–D). Therefore, the co-culture pattern B4-L had the greatest ability to transform the rice straw to SCC.

There are many studies have documented that the degraded ratio and rate of co-culture of fungi were significantly higher than the culture of single fungus^9,13^. Although we did not compare the digestibility of co-culture of *P. chrysosporium* and *T. viride*, and that of single *P. chrysosporium* or *T. viride*, the degradation ratio of B5-L significantly lower than that of B4-L (Fig. 2A–D) implied that the digestibility of single *P. chrysosporium* was probably lower than that of the B4-L.

### Firstly inoculated *P. chrysosporium* and cultured for 4 days, and subsequently inoculated *T. viride* and then co-cultured for 4 days (B4-L4) was the optimum co-culture time pattern

Although our results showed the optimum co-culture pattern was first inoculation of *P. chrysosporium* and culture for 4 days, followed by inoculation of *T. viride*, and then co-culture for 3 days, the optimum co-culture time after the inoculation of *T. viride* was still unclear. Theoretically, longer co-culture time would increase DRL and DRC. However, increasing co-culture time would cause higher energy consumption, and probably reduced SCC in the culture product. Therefore, it is important to screen the optimum co-culture time according to DRL and DRC, and SCC in culture product. Five groups were established to analyze the optimum co-culture time, i.e. B4-L2, B4-L3, B4-L4, B4-L5, and B4-L6. Three repetitive samples were set in each group. There was no significant difference in cellulase activity among the groups with exception to B4-L6 (Fig. 2E). DRL and DRC of the groups B4-L4 and B4-L5 were higher than other groups (Fig. 2F,G), and SCC in the culture product of the group B4-L4 was higher than other groups (Fig. 2H). The results showed that the best co-culture time pattern was B4-L4, i.e. firstly inoculated *P. chrysosporium* and cultured for 4 days, and subsequently inoculated *T. viride* and then co-cultured for 4 days.

### Equivalent inoculation of *P. chrysosporium* and *T. viride* was the optimum inoculation proportion

Inoculation proportion of co-cultured fungi influences their growth and metabolic activities. To analyze the optimum inoculation proportion of *P. chrysosporium* (B) and *T. viride* (L), the degradation efficiencies of seven inoculation proportions, i.e. 1:4, 3:7, 2:3, 1:1, 3:2, 7:3, and 4:1 (B/L), were compared. The cellulase activity was the highest when the inoculation proportion was 1:1. Simultaneously, DRL and DRC were significantly higher than those in other inoculation proportions, and SCC in the culture product was also the highest (Fig. 2I–L). Although *P. chrysosporium* was firstly inoculated and cultured 4 days, our results showed that because *T. viride* had an obviously competitive advantage, *T. viride* could quickly reproduce and participate in the degradation reaction of the rice straw. However, the ratio of *P. chrysosporium* and *T. viride* in the final culture product should be further measured in future study.

### Fourteen percent fungal solution was the optimum inoculum concentration

Inoculum concentration influences the initial growth of fungi and the degradation efficiency. Our results showed that the optimum inoculum concentration was 14% (w/v), with 32.46% of DRC, 26.38% of DRL, and 22.40% of SSC in the culture product (Figs. 2M–P). In addition, although there was no significantly difference, the degradation efficiency of rice straw was slightly lower when the inoculum concentration was 12% or 16% (Figs. 2M–P). Theoretically, if the inoculated fungi directly degraded the rice straw, the higher inoculum concentration could cause the higher degradation efficiency. However, our results showed that when the inoculum concentration was 16%, the degradation efficiency of rice straw, and the SCC was slightly reduced. These results implied that the inoculated fungi did not degrade the rice straw directly, but experienced an unknown regulatory process.

### The optimum solid-liquid ratio was 1:1.4

DRL and DRC increased with the decrease of solid-liquid ratio from 1:1 to 1:1.4, but the degradation ratio decreased when the solid-liquid ratio was less than 1:1.4. The degradation ratio was the highest when the solid-liquid ratio was 1:1.4. DRL and DRC were 26.07% and 33.11%, respectively. The SCC in the culture product was 23.70% (Fig. 2Q–T).

### The optimum culture temperature was 30 °C

DRL and DRC increased with the increase of culture temperature when the temperature was lower than 30 °C, but they decreased when the temperature was higher than 30 °C (Fig. 2U–X). The optimum temperature was 30 °C. At the temperature, the DRL (26.38%) and DRC (33.29%) were the highest. And the SCC in the culture product was the highest (23.07% (w/v); Fig. 2U–X).

Although there were some reports about the stepwise co-culture of fungi to degrade rice straw^14^, the DRLs and DRCs were commonly less than 30%, and the culture cycle was commonly more than 10 days. In addition, although some anaerobic fungi from rumen can degrade the rice straw reaching to the digestibility of 50%^15^, the reaction time and the SCC in the culture product should be considered. Because we did not just degrade the rice straw, we actually using the fungi to transform the rice straw to more accessible energy resources, such as ethanol and glucose. In the present study, under the optimum condition, the DRL and DRC reached to 26.38% and 33.29% during 8 days, respectively.

## Conclusions

The best co-culture pattern of the fungi was B4-L4, i.e. firstly inoculated *P. chrysosporium* and cultured for 4 days, and subsequently inoculated *T. viride* and co-cultured for 4 days. The optimum co-culture condition was 14% (w/v) of the inoculum concentration, the equivalent inoculation of the fungi, 30 °C of the culture temperature, and 1:1.4 of the solid-liquid ratio. Under the optimum co-culture condition, the DRL and DRC were 26.38% and 33.29%, respectively; and the SCC in the culture product was 23.07% (w/v).

## Materials and Methods

### Experimental design, microorganism strains, and culture conditions

*T. viride* ACCC30169 and *P. chrysosporium* ACCC30942 were provided by microbiology laboratory of College of Bioscience and Biotechnology, Hunan Agricultural University. *T. viride* exists high ability to degrade cellulose, and *P. chrysosporium* exists high ability to degrade lignin. The strains stored at 4 °C were inoculated to fresh PDA slant culture-medium (The 20% (w/v) of peeled potato blocks were boiled for 20 min and filtered. 2% (w/v) of glucose, and 2% (w/v) of agar was added to the filtrate and its pH was regulated to 7.2; then the culture medium was steam-sterilized at 121 °C to 25 min using a high-pressure steam sterilizer.), then the inoculations were cultured for 5 days at 28 °C. Subsequently, the spores of *T. viride* and *P. chrysosporium* were picked from the PDA slant culture-medium by oese and inoculated to 100 ml of seed liquid medium (The seed liquid medium contains 2% (w/v) of wheat bran, 2% (w/v) of glucose, 2% (w/v) of bean cake powder, 1% (w/v) of sucrose, 0.5% (w/v) of (NH_4_)_2_SO_4_, 0.2% (w/v) of KH_2_PO_4_, 0.1% (w/v) of MgSO_4_·7H_2_O, and 0.2% (w/v) of yeast powder. Its pH was regulated to 7.2; then the culture medium was steam-sterilized at 121 °C to 25 min using a high-pressure steam sterilizer.) in the 250 ml conical flasks, and cultured for 3 days at 28 °C with 150r/min of shake. Ten percent (V/W) of seed liquid mediums were inoculated to the solid fermentation medium, mixed and cultured for 7 days at 28 °C in a constant temperature incubator with mixed once per 24 hours. The solid fermentation medium contains 85% of straw comminution (The rice straw was provided by the Rice Experimental Field of Human Agricultural University. The rice straw was cut to 2–3 cm of fragments and dried to less than 5% of moisture under 80 °C. Then the dried straw fragments were shattered to less than 10 mesh.), 10% of wheat bran, 2.5% of glucose, 2% of bean cake powder, 0.4% of KH_2_PO_4_, and 0.1% of MgSO_4_·7H_2_O. Its pH was regulated to 7.2; then the culture medium was steam sterilized at 121 °C to 25 min using a high-pressure steam sterilizer.

To obtain the optimum co-culture condition of *P. chrysosporium* and *T. viride* co-culture to rice straw, we firstly tested the antagonistic characteristic between the fungi, and then optimized the co-culture conditions through optimizing the co-culture pattern, fermentation time, inoculation proportion of the fungi, inoculum concentration, solid-liquid ratio, and culture temperature.

### Antagonistic experiment

The spores of the fungi were picked from the PDA slant culture-medium by oese and inoculated to two poles of PDA solid plate medium. The inverted plates were cultured at 28 °C in a constant temperature incubator and the growth of fungi was observed every day.

### Co-culture pattern and time experiments

To screen the optimum co-culture pattern of the fungi to degrade the rice straw, we set eleven combination groups as shown in Table 1. Ten percent of seed culture medium (v/w) was inoculated to solid fermentation medium and mixed, then cultured at 28 °C in a constant temperature incubator. It was stirred once a day in all fermentation experiments.

**Table 1:** Experimental co-culture patterns of *T. viride* and *P. chrysosporium*.

Co-culture pattern	Firstly inoculated and cultured days	Secondly inoculated and co-cultured days
B-L	*T. viride* and *P. chrysosporium* synchronously	Co-cultured 7 days
B1-L	*P. chrysosporium*, 1 day	*T. viride*, co-cultured 6 days
B2-L	*P. chrysosporium*, 2 days	*T. viride*, co-cultured 5 days
B3-L	*P. chrysosporium*, 3 days	*T. viride*, co-cultured 4 days
B4-L	*P. chrysosporium*, 4 days	*T. viride*, co-cultured 3 days
B5-L	*P. chrysosporium*, 5 days	*T. viride*, co-cultured 2 days
L1-B	*T. viride*, 1 days	*P. chrysosporium*, co-cultured 6 days
L2-B	*T. viride*, 2 days	*P. chrysosporium*, co-cultured 5 days
L3-B	*T. viride*, 3 days	*P. chrysosporium*, co-cultured 4 days
L4-B	*T. viride*, 4 days	*P. chrysosporium*, co-cultured 3 days
L5-B	*T. viride*, 5 days	*P. chrysosporium*, co-cultured 2 days

The total culture time was 7 days. The co-culture patterns were consisted of the firstly inoculated fungus (B represented *P. chrysosporium* and L represented *T. viride*) and cultured days (from 1 to 5), and the secondly inoculated fungus. For instance, B2-L represented that firstly inoculated *P. chrysosporium* and cultured 2 days, and then inoculated *T. viride* and co-cultured 5 days. B-L represented that inoculated *T. viride* and *P. chrysosporium* synchronously and co-cultured 7 days.

To screen the optimum co-culture time of the fungi to degrade the rice straw, the optimum co-culture pattern of the fungi was used, i.e., the culture time of firstly inoculated *P. chrysosporium* was selected according to the result of the co-culture pattern experiments, and the co-culture time after inoculated *T. viride* was changed from two to six days. The solid fermentation mediums were cultured at 28 °C in a constant temperature incubator.

### Inoculation proportion and inoculum concentration experiments

To screen the optimum inoculation proportion of the fungi culture to degrade the rice straw, the optimum co-culture pattern of the fungi and the optimum co-culture time was used. A series of inoculation proportions of the fungi, i.e. 1:4, 3:7, 2:3, 1:1, 3:2, 7:3, and 4:1 were compared. The solid fermentation mediums were cultured at 28 °C in a constant temperature incubator.

To screen the optimum inoculum concentration of the co-culture to degrade the rice straw, the optimum co-culture pattern, the optimum co-culture time, and the optimum inoculation proportion of the fungi were used. A series of inoculum concentrations (w/v), i.e. 8%, 10%, 12%, 14%, and 16% were compared. The solid fermentation mediums were cultured at 28 °C in a constant temperature incubator.

### Solid-liquid ratio and culture temperature experiments

To screen the optimum solid-liquid ratio of the co-culture to degrade the rice straw, the optimum co-culture pattern, co-culture time, inoculation proportion, and inoculum concentration of the fungi was used. A series of solid-liquid ratios, i.e. 1:1, 1:1.2, 1:1.4, 1:1.6, and 1:1.8 were compared. The solid fermentation mediums were cultured at 28 °C in a constant temperature incubator.

To screen the optimum co-culture temperature of the co-culture to degrade the rice straw, the optimum co-culture pattern, co-culture time, inoculation proportion, inoculum concentration, and the solid-liquid ratio of the fungi was used. A series of culture temperatures, i.e. 24, 26, 28, 30, and 32 °C were compared. The solid fermentation mediums were cultured in constant temperature incubators.

### Determination of enzyme activities and polysaccharide contents

Two grams of culture products were accurately weighed and added to 50 ml of distilled water, and then were extracted to crude enzymes at 30 °C with 150 r/min of shake. The filter paper enzyme activity (FPA) and CMC enzyme activity (CMCase) were used to indicate the cellulase activity. Endoglucanase and filter paper enzyme activities in the crude enzyme extracts were determined using 3,5-dinitrosalicylic acid (DNS) method^16^. Cellulose and lignin contents were determined according to previous reports^17,18^. DRL and DRC were calculated according to previous reports^19,20^. SCC was determined referenced to a previous report^21^.

### Data analysis

Results for each parameter are presented as the mean ± standard error (SE) for each group. One-way analysis of variance (ANOVA) was used for testing difference among different treatments, and Tukey’s honestly significant difference (Tukey’s HSD) method was used to the post-hoc test. All statistical analyses were conducted using R version 3.5.1. P-values < 0.05 were considered statistically significant.
